# Case report: A novel pathogenic FRMD7 variant in a Turner syndrome patient with familial idiopathic infantile nystagmus

**DOI:** 10.3389/fneur.2023.1199095

**Published:** 2023-07-20

**Authors:** Sara Hafdaoui, Claudia Ciaccio, Barbara Castellotti, Francesca L. Sciacca, Chiara Pantaleoni, Stefano D'Arrigo

**Affiliations:** ^1^Department of Pediatric Neurosciences, Fondazione IRCCS Istituto Neurologico Carlo Besta, Milan, Italy; ^2^Department of Medical Genetics and Neurogenetics, Fondazione IRCCS Istituto Neurologico Carlo Besta, Milan, Italy; ^3^Laboratory of Cytogenetic, Neurological Biochemistry and Neuropharmacology Unit, Department of Diagnostic and Technology, Fondazione IRCCS Istituto Neurologico Carlo Besta, Milan, Italy

**Keywords:** FRMD7, idiopathic nystagmus, Turner syndrome, X-linked IIN, X-linked nystagmus

## Abstract

Infantile idiopathic nystagmus (IIN) is an oculomotor disorder characterized by involuntary bilateral, periodic ocular oscillations, predominantly on the horizontal axis. X-linked IIN (XLIIN) is the most common form of congenital nystagmus, and the FERM domain-containing gene (*FRMD7*) is the most common cause of pathogenesis, followed by mutations in *GPR143*. To date, more than 60 pathogenic *FRMD7* variants have been identified, and the physiopathological pathways leading to the disease are not yet completely understood. *FRMD7*-associated nystagmus usually affects male patients, while it shows incomplete penetrance in female patients, who are mostly asymptomatic but sometimes present with mild ocular oscillations or, occasionally, with clear nystagmus. Here we report the first case of a patient with Turner syndrome and INN in an XLIIN pedigree, in which we identified a novel frameshift mutation (c.1492dupT) in the FRMD7 gene: the absence of one X chromosome in the patient unmasked the presence of the familial genetic nystagmus.

## 1. Introduction

The *FRMD7* gene is located in the Xq26.2 chromosomal region; it consists of 12 exons encoding a 714-residue protein, FRMD7, whose cellular function is still debated (1–3). It is known that the FRMD7 protein contains a FERM domain at the N-terminus, indicating its possible participation in signal transduction between the cell membrane and the cytoskeleton (4), similar to other FERM domain proteins (5, 6).

The gene is expressed in various tissues, namely in the brain areas responsible for eye movement control (such as the midbrain and cerebellum) and the retina. Studies in mice have detected *FMRD7* mRNA in the ventricular layer of the forebrain, suggesting that the protein plays a role in the development of nerve cells in these areas of the brain and retina (7, 8).

More than 60 *FRMD7* variants have been described in X-linked infantile nystagmus, most of them missense variants; pathogenic variants of the gene are likely to result in the production of an unstable protein that is unable to perform its normal functions, therefore disrupting nerve cell development in the expected areas of influence (2, 7). This malfunctioning of the brain areas that control eye movements, along with retinal misdevelopment, is thought to cause the involuntary side-to-side eye movements that are characteristic of X-linked infantile nystagmus (7, 9, 10).

Genetic studies of nystagmus are increasingly being reported, expanding the clinical and molecular knowledge of this disorder.

Turner syndrome is a chromosomal abnormality caused by the deletion or non-functioning of one X chromosome in a phenotypically female individual. In about 50% of cases, it is caused by an X monosomy (45,X0 karyotype), while the other half are mosaic patients carrying an X monosomy component and a normal component (45,X0/46,XX or 45,xo/46,XY); in addition, in rare cases, Turner syndrome can result from peculiar chromosomal abnormalities leading to a non-functional X chromosome, such as isochromosome Xq (an X chromosome composed of two copies of the long arm of the X chromosome linked together), ring X with partial loss of genetic material in both the long and short arms, or Xp/ Xq deletions (11, 12). With an incidence of 1/2,500 births, it is one of the most common chromosomal anomalies observed. Two main random pathogenic mechanisms are known to cause the disorder: a nondisjunction event occurring during germ cell development, with the creation of an egg or sperm cell lacking an X chromosome, which, together with a normal germ cell, generates an embryo with an anomalous number of chromosomes and an error during the cell division cycles of early fetal development, resulting in a mosaic asset with a part of cells with a normal karyotype and a part of cells with an X0 alteration (11). It is typically a *de novo* condition, because most patients are infertile, but in extremely rare cases it can be transmitted from one generation to the next (12). The phenotype of Turner syndrome arises from X-linked genes that escape inactivation: short stature and Madelung's deformity result from mutations in the *SHOX* gene (13), while gonadal dysgenesis involves genes such as *USP9X, RPS4X*, and *DIAPH2* (12, 14).

The X chromosome also contains several genes responsible for X-linked disorders, which typically manifest in male subjects and are masked in female subjects by the presence of a normally functioning gene on the other X. The absence of an X chromosome may therefore expose Turner's patients to the occurrence of familial genetic disorders that do not usually affect female individuals.

## 2. Case presentation

**Figure 1:**
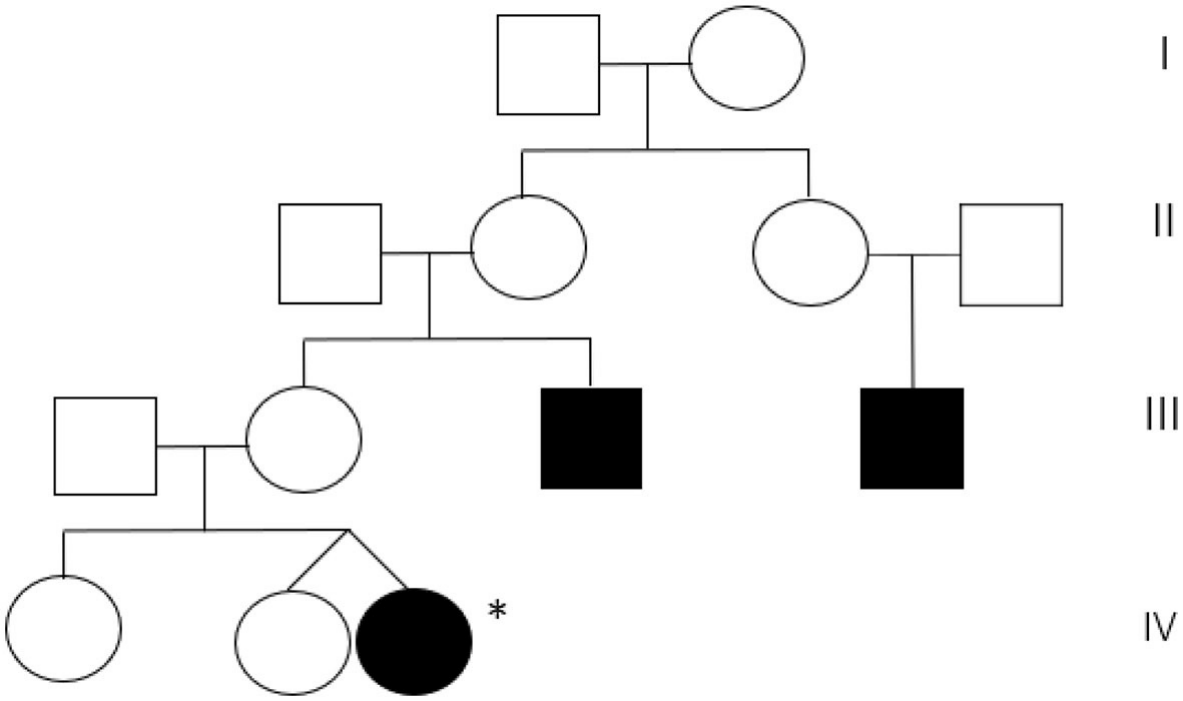
Maternal pedigree of the patient. Proband is marked with an asterisk.

A 5-month-old girl was referred to our center because of early-onset nystagmus. Her family history was positive for nystagmus, present in the maternal uncle and a male cousin of the mother (Figure 1).

The girl was born from a heterozygous twin pregnancy; her female twin and an older sister were reported to be in good general health, as were her parents. The pregnancy was complicated by gestational hypertension, and a cesarean section was performed at 35 + 3 weeks gestation. At birth, she showed normal growth parameters (weight 2,070 g, length 47 cm, head circumference 31 cm) and no perinatal distress (Apgar score 10/10), but she later experienced poor sucking for a few days and an anterior ectopic anus was detected.

At the age of 3 months, her father started noticing anomalous horizontal eye movements; the girl underwent an ophthalmologic examination, which confirmed the presence of a pendular nystagmus of low frequency and good amplitude, without convergence problems or fundus oculi alterations.

Clinical examination revealed good general health and normal growth (length 62 cm =25–50°, weight 6.3 kg = 25–50°, head circumference 40.5 cm = 10–25°). Dysmorphic facial features were present (bitemporal narrowing, epicanthus, simplified and protruding ears), in addition to mild telethelia, inverted nipples, the previously reported anteriorly displaced anus, a hairless sacral dimple, and a congenital melanocytic nevus on the right leg covered with hair and measuring 6 × 3 cm. Neurological examination showed bilateral pendular horizontal nystagmus, mildly increased muscular tone, and mild motor developmental delay.

The patient underwent several diagnostic examinations, including routine blood work, metabolic screening (plasma amino acids and urine organic acids), brain MRI, EEG, and evoked potentials (VEP, ERG, BAEP), all with normal results; in particular, brain MRI showed no signs of optic nerve atrophy, and VEP and ERG showed no alterations. Once again, the girl underwent an ophthalmologic evaluation, which confirmed normal fundus oculi, no photophobia, and no additional pathologic findings other than the nystagmus.

All the data pointed to a genetic condition. Array CGH was performed with an ISCA resolution of 19 kb, with the following result: Xp22.33q28 (16961_155208387)x1, meaning a deletion of an entire heterosome. Therefore, we also asked for a karyotype, performed on 16 metaphases with a resolution of 550 bands, which confirmed an X monosomy (Turner syndrome) in all analyzed metaphases. Nystagmus is an uncommon feature in Turner's patients, with a variable prevalence depending on the study, ranging from 4 up to 20% (15, 16). Given the family history and the genotype of the baby, we hypothesized that the nystagmus could be a symptom of an X-linked condition caused by the loss of one of the sex chromosomes and, in particular, an alteration in the *FRMD7* gene.

Sequencing of the *FMRD7* gene was performed by NGS technology and highlighted the presence of a hemizygous frameshift variant (c.1492dupT), which determines the production of an aberrant and prematurely truncated protein (p.Tyr498Leufs^*^15), and is classified as probably pathogenic according to the ACMG guidelines (17) (PVS1, PM2 criteria). The same variant was detected in the healthy mother, while the male relatives, living abroad, were not available for testing. The nystagmus was therefore confirmed to be caused by this mutation, which was revealed by Turner syndrome.

## 3. Discussion

45,X0 is the most common karyotype in patients with Turner syndrome, accounting for ~50% of all cases (12, 18). Typical manifestations of the condition include short stature, hypogonadism, and/or other types of gonadal dysplasia (often with primary amenorrhea), a webbed neck, mammary hypoplasia with spaced and inverted nipples, heart malformations (most commonly bicuspid aortic valve, aortic coarctation, and aortic valvulopathy), and skeletal (shield-like chest, cubitus valgus, scoliosis) or genitourinary abnormalities (horseshoe kidney, anus imperforatus, anal atresia) (11, 12, 18). The phenotype can be very different among patients and mostly depends on the karyotype, with X0 individuals having the most severe presentation and mosaic individuals showing severity and gonadal differentiation depending on the ratio of 45,X0/normal cells (12, 18).

Ophthalmologic defects are not typical of Turner syndrome, and the most common features emerging from the few available studies are ametropia and strabismus, both of which are also known to be common in the general pediatric population (19). Nystagmus has been listed in the group of “uncommon ophthalmological defects” (prevalence 5%−25%) in a paper by Denniston and Butler (15) and estimated at 4% in a study by Wikiera et al. (16).

In our case, the family history was suggestive of hereditary nystagmus, given the presence of two affected male relatives in the maternal line.

*FRMD7* variants are among the most common causes of hereditary nystagmus.

The gene was first suspected to be involved in the condition in 2006, following a work published by Tarpey et al. (3) that identified an *FMRD7* variant in 15 of 16 families with congenital nystagmus. The gene is now known to be a member of the protein 4.1 superfamily and to have a highly conserved NH2-terminus containing the B41 and the FERM-C domains (20–22). The FERM domain at the N-terminus is also present in other proteins, such as FARP1 and FARP2, and studies in rats have demonstrated that it modulates the length and branching of neurites in embryonic cortical neurons and reorganizes the cytoskeleton (7, 21). In adult humans, the FRMD7 protein is absent in ocular tissues but has been detected during embryonic stages in the developing neural retina and in brain regions that control eye movements (forebrain, midbrain, cerebellar primordium) (3–6). Recently, foveal hypoplasia and developmental abnormalities of the optic nerve head have been reported in patients with *FRMD7* pathogenic variants as a result of retinal neuronal migratory disorders due to impaired growth cone guidance, which is consistent with the expression patterns observed in the developing retina and optic nerve (23). The dysfunction of FRMD7 may contribute to the absence of the horizontal optokinetic reflex through the loss of horizontal direction selectivity. These findings suggest that the abnormal development of the afferent visual system may be associated with *FRMD7* variants and may affect neural circuits within the oculomotor system, leading to abnormal eye movements and gaze instability (22). Taken together, these data provide strong evidence that the FRMD7 protein plays a role in the neural development of visual circuits.

Although we did not perform a functional analysis of the protein, a review of the FRMD7 literature shows that in mice, null mutations in FRMD7 alter neurite length and the branching process of neurons in the midbrain, cerebellum, and retina. This is a plausible explanation for how defects in the protein coded by *FRMD7* cause disease (4, 24).

FRMD7-related infantile nystagmus is characterized by either the onset of horizontal, conjugate, gaze-dependent, or time-dependent nystagmus in the first 6 months of life or periodic alternating nystagmus (with cyclic changes in nystagmus direction) with infantile onset. Binocular vision and color vision are normal, and visual acuity is usually good (>6/12). In total, 15% of affected individuals have an abnormal head posture as the consequence of a continuous attempt to reach an eccentric null point (a point of gaze where oscillation is minimally present) (25, 26). The optokinetic response is abnormal, and both low gains and reversal patterns have been described (25). No particular genotype-phenotype has been described regarding such ophthalmologic features; indeed, studies have shown extensive intra- and interfamilial variability in the clinical presentation (8, 25, 27).

In our case, once the patient was found to have Turner syndrome, the most likely condition to explain her nystagmus was an *FMRD7* alteration. Considering other XL conditions associated with nystagmus, the little girl did not show the iris hypopigmentation that is usual in type 1 ocular albinism; there were no symptoms or signs of photophobia present, as expected in blue cone monochromatism; and dark adaptation was normal, thus ruling out congenital stationary night blindness.

*FRMD7* sequencing was therefore performed, confirming the presence of the pathogenic variant c.1492dupT (p.Tyr498LeufsTer15) that, consistent with family history, was inherited from her healthy mother.

XLIIN shows an incomplete penetrance in carrier females (27–29), probably as a consequence of the skewed X inactivation pattern, resulting in an unbalanced inactivation of the paternal and maternal X chromosomes established in embryonic life (28–30).

In our family, we did not study the X inactivation pattern and FRMD7 testing was not performed in the two patient siblings for ethical reasons (they are both healthy minors).

## 4. Conclusions

We presented the case of a girl with a phenotype mimicking that of a more severe condition and found it to be the consequence of a double diagnosis of Turner syndrome plus familial *FRMD7*-related nystagmus.

Given the phenotype, the first diagnostic hypothesis was that a single disease could justify all the clinical features of the girl. Array-CGH analysis revealed the diagnosis of Turner syndrome, which explains almost all the symptoms (spaced and inverted nipples, anal anomaly, etc.) but not the nystagmus. Moreover, the maternal family history was positive for nystagmus in male relatives, which is extremely relevant to anamnestic data. *FRMD7*-related nystagmus was confirmed by the targeted molecular analysis, which identified the maternal frameshift variant in exon 12 c.1492dupT (p.Tyr498LeufsTer15), not previously described and predicted *in silico* to be pathogenic. Other frameshift variants in the same exon are described and analyzed in different studies, highlighting the important role of the highly conserved C-terminal region of FRMD7 (31, 32). These findings lead us to the diagnosis of *FRMD7* X-linked nystagmus with Turner syndrome.

With the advent of NGS, we have access to extremely sophisticated genetic methods that have allowed us to make great strides in the knowledge of genetics and pathologies; in fact, international genetic guidelines recommend WES as a first-step analysis in the case of psychomotor delay and intellectual disability. However, it is useful to remember that the techniques of classical and molecular cytogenetics must not be abandoned, as in this case they led to a simplified diagnostic algorithm.

In conclusion, our results broaden the mutation spectrum of FRMD7. Finally, this work highlights the importance of a sequential and precise diagnostic algorithm that, starting from a careful collection of anamnestic data and clinical examination, facilitates the achievement of a diagnosis without bias or waste of resources.

## Data availability statement

The datasets presented in this article are not readily available because of ethical and privacy restrictions. Requests to access the datasets should be directed at: the corresponding author.

## Ethics statement

Ethical review and approval was not required for the study on human participants in accordance with the local legislation and institutional requirements. Written informed consent to participate in this study was provided by the participants' legal guardian/next of kin. Written informed consent was obtained from the individual(s), and minor(s)' legal guardian/next of kin, for the publication of any potentially identifiable images or data included in this article.

## Author contributions

SH: patient evaluation and manuscript writing. CC: patient evaluation, manuscript writing, and editing. BC and FLS: genetic testing performance and manuscript revision. CP and SD: supervision, manuscript revision, and editing. All authors had access and approved the final version of the manuscript.

## References

[1] Zhao H Huang X-F Zheng Z-L Deng W-L Lei X-L Xing D-J. Molecular genetic analysis of patients with sporadic and X-linked infantile nystagmus. BMJ Open. (2016) 6:e010649. 10.1136/bmjopen-2015-010649 27036142 PMC4823450

[2] Chen J Wei Y Tian L Kang X. A novel frameshift mutation in FRMD7 causes X-linked infantile nystagmus in a Chinese family. BMC Med Genet. (2019) 20:5. 10.1186/s12881-018-0720-8 30616528 PMC6323710

[3] Tarpey P Thomas S Sarvananthan N Mallya U Lisgo S Talbot CJ. Mutations in FRMD7, a newly identified member of the FERM family, cause X-linked idiopathic congenital nystagmus. Nat Genet. (2006) 38:1242–4. 10.1038/ng1893 17013395 PMC2592600

[4] Betts-Henderson J Bartesaghi S Crosier M Lindsay S Chen H-L Salomoni P. The nystagmus-associated FRMD7 gene regulates neuronal outgrowth and development. Hum Mol Genet. (2010) 19:342–51. 10.1093/hmg/ddp500 19892780

[5] Cho W Stahelin RV. Membrane-protein interactions in cell signalling and membrane trafficking. Annu Rev Biophys Biomol Struct. (2005) 34:119–51. 10.1146/annurev.biophys.33.110502.133337 15869386

[6] Sun CX Robb VA Gutmann DH. Protein 4, 1. tumour suppressors: getting a FERM grip on growth regulation. J Cell Sci. (2002) 115(Pt 21):3991–4000. 10.1242/jcs.00094 12356905

[7] Watkins RJ Thomas MG Talbot CJ Gottlob I Shackleton S. The role of FRMD7 in Idiopathic Infantile Nystagmus. J Ophtalmol. (2012) 460957. 10.1155/2012/460956 21904664 PMC3163398

[8] Self J Haitchi HM Griffiths H Holgate ST Davies DE Lotery A. Frmd7 expression in developing mouse brain. Eye. (2010) 24:165–9. 10.1038/eye.2009.44 19265863

[9] Pu J Li Y Liu Z Yan Y Tian J Chen S. Expression and localization of FRMD7 in human fetal brain, and a role for F-actin. Mol Vis. (2011) 17:591–7. 21386928 PMC3049738

[10] Pu J Mao Y Lei X Yan Y Lu X Tian J. FERM domain containing protein interacts with the Rho GDP Dissociation inhibitor and specifically activates Rac1 signalling. PLoS ONE. (2013) 8:e73108. 10.1371/journal.pone.0073108 23967341 PMC3742540

[11] Ranke MB Saenger P. Turner's syndrome. Lancet. (2001) 28:358. 10.1016/S0140-6736(01)05487-3 11498234

[12] Shankar Kikkeri N Nagalli S. Turner's Syndrome. [Updated 2022 Jun 20]. In: StatPearls [Internet]. Treasure Island, FL: StatPearls Publishing (2022).

[13] Ogata T Matsuo N Nishimura G. SHOX haploinsufficiency and overdosage: impact of gonadal function status. J Med Genet. (2001) 38:1–6. 10.1136/jmg.38.1.1 11134233 PMC1734713

[14] Yuan X Zhu Z. Turner syndrome with rapidly progressive puberty: a case report and literature review. J Int Med Res. (2020) 48:300060519896914. 10.1177/0300060519896914 32357117 PMC7221220

[15] Denniston AKO Butler L. Ophthalmic features of Turner's syndrome. Eye. (2004) 18:680–4. 10.1038/sj.eye.6701323 15002027

[16] Wikiera B Mulak M Koltowska-Haggstrom M Noczynska A. The presence of eye defects in patients with Turner syndrome is irrespective of their karyotype. Clin Endocrinol. (2015) 83:842–8. 10.1111/cen.12794 25871912

[17] Richards S Aziz N Bale S Bick D Das S Gastier-Foster J. Standards and guidelines for the interpretation of sequence variants: a joint consensus recommendation of the American College of Medical Genetics and Genomics and the Association for Molecular Pathology. Genet Med. (2015) 17:405–24. 10.1038/gim.2015.30 25741868 PMC4544753

[18] Sybert VP McCauley E. Turner's syndrome. N Engl J Med. (2004) 351:1227–38. 10.1056/NEJMra030360 15371580

[19] Huang J Basith SST Patel S Goetsch Weisman A Brickman W Mets MB. Ocular findings in paediatric Turner syndrome. Ophthalmic Genet. (2022) 43:450–3. 10.1080/13816810.2022.2045512 35382690

[20] Li N Wang L Cui L Zhang L Dai S Li H. Five novel mutations of the FRMD7 gene in Chinese families with X-linked infantile nystagmus. Mol Vis. (2008) 14:733–8. 18431453 PMC2324116

[21] Baines AJA. A FERM-adjacent (FA) region defines a subset of the 4.1 superfamily and is a potential regulator of FERM domain function. BMC Genomics. (2006) 7:85. 10.1186/1471-2164-7-85 16626485 PMC1459144

[22] Du W Bu J Dong J Jia Y Li J Liang C. A novel frame-shift mutation in FRMD7 causes X-linked idiopathic congenital nystagmus in a Chinese family. Mol Vis. (2011) 17:2765–8. 22065930 PMC3209434

[23] Thomas MG Crosier M Lindsay S Kumar A Araki M Leroy BP. Abnormal retinal development associated with FRMD7 mutation. Hum Mol Genet. (2013) 22:4086–93. 10.1093/hmg/ddu122 24688117 PMC4082370

[24] Pu J Lu X Zhao G Yan Y Tian J Zhang B. FERM domain containing protein 7 (FRMD7) upregulates the expression of neuronal cytoskeletal proteins and promotes neurite outgrowth in Neuro-2a cells. Mol Vis. (2012) 18:1428–35. 22690121 PMC3370689

[25] Thomas S Proudlock FA Sarvananthan N Roberts EO Awan M McLean R. Phenotypical characteristics of idiopathic infantile nystagmus with and without mutations in FRMD7. Brain. (2008) 131:1259–67. 10.1093/brain/awn046 18372314

[26] Thomas MG Crosier M Lindsay S Kumar A Thomas S Araki M. The clinical and molecular genetic features of idiopathic infantile periodic alternating nystagmus. Brain. (2011) 134:892–902. 10.1093/brain/awq373 21303855 PMC4125620

[27] Shiels A Bennett TM Prince JB Tychsen L. X-linked idiopathic infantile nystagmus associated with a missense mutation in FRMD7. Molec Vis. (2007) 13:2233–41. 18087240

[28] Kaplan Y Vargel I Kansu T Akin B Rohmann E Kamaci S. Skewed X inactivation in an X linked nystagmus family resulted from a novel, p.R229G, missense mutation in the FRMD7 gene. Br J Ophthalmol. (2008) 92:135–41. 10.1136/bjo.2007.128157 17962394

[29] Zhang B Liu Z Zhao G Xie X Yin X Hu Z. Novel mutations of the FRMD7 gene in the X-linked congenital motor nystagmus. Mol Vis. (2007) 13:1674–9. 17893669

[30] Pereira G Dória S. X-chromosome inactivation: implications in human disease. J Genet. (2021) 100:63. 10.1007/s12041-021-01314-1 34553695

[31] Schouten JP McElgunn CJ Waaijer R Zwijnenburg D Diepvens F Pals G. Relative quantification of 40 nucleic acid sequences by multiplex ligation-dependent probe amplification. Nucleic Acid Res. (2002) 30:e57. 10.1093/nar/gnf056 12060695 PMC117299

[32] AlMoallem B Bauwens M Walraedt S Delbeke P De Zaeytijd J Kestelyn P. Novel FRMD7 mutations and genomic rearrangement expand the molecular pathogenesis of X-linked idiopathic infantile nystagmus. Invest Ophtalmol Vis Sci. (2015) 56:1701–10. 10.1167/iovs.14-15938 25678693

